# Combined PDGFR and HDAC Inhibition Overcomes PTEN Disruption in Chordoma

**DOI:** 10.1371/journal.pone.0134426

**Published:** 2015-08-06

**Authors:** Dae-Hee Lee, Ying Zhang, Amin B. Kassam, Myung-Jin Park, Paul Gardner, Daniel Prevedello, Stephanie Henry, Craig Horbinski, Jan H. Beumer, Hussein Tawbi, Brian J. Williams, Mark E. Shaffrey, Merrill J. Egorin, Roger Abounader, Deric M. Park

**Affiliations:** 1 Department of Neurological Surgery, University of Virginia, PO BOX 800212, Charlottesville, VA 22908, United States of America; 2 Department of Microbiology, University of Virginia, PO BOX 800734, Charlottesville, VA 22908, United States of America; 3 Department of Neurological Surgery, University of Pittsburgh, 200 Lothrop Street, Pittsburgh, PA 15213, United States of America; 4 Department of Pathology, University of Pittsburgh, 200 Lothrop Street, Pittsburgh, PA 15213, United States of America; 5 University of Pittsburgh Cancer Institute, University of Pittsburgh, 5150 Centre Avenue, Pittsburgh, PA 15232, United States of America; 6 Neuro-Oncology Branch, National Cancer Institute, NIH, 9030 Old Georgetown Rd, B82/Rm225, Bethesda, MD 20892, United States of America; Johns Hopkins University, UNITED STATES

## Abstract

**Background:**

The majority of chordomas show activation of the platelet-derived growth factor receptor (PDGFR). Based on *in vitro* intertumoral variation in response to recombinant PDGF protein and PDGFR inhibition, and variable tumor response to imatinib, we hypothesized that chordomas resistant to PDGFR inhibition may possess downstream activation of the pathway.

**Methods:**

Molecular profiling was performed on 23 consecutive chordoma primary tissue specimens. Primary cultures established from 20 of the 23 specimens, and chordoma cell lines, UCH-1 and UCH-2, were used for *in vitro* experiments.

**Results:**

Loss of heterozygosity (LOH) at the phosphatase and tensin homolog (*PTEN*) locus was observed in 6 specimens (26%). *PTEN* disruption statistically correlated with increased Ki-67 proliferation index, an established marker of poor outcome for chordoma. Compared to wild type, PTEN deficient chordomas displayed increased proliferative rate, and responded less favorably to PDGFR inhibition. *PTEN* gene restoration abrogated this growth advantage. Chordomas are characterized by intratumoral hypoxia and local invasion, and histone deacetylase (HDAC) inhibitors are capable of attenuating both hypoxic signaling and cell migration. The combination of PDGFR and HDAC inhibition effectively disrupted growth and invasion of PTEN deficient chordoma cells.

**Conclusions:**

Loss of heterozygosity of the *PTEN* gene seen in a subset of chordomas is associated with aggressive *in vitro* behavior and strongly correlates with increased Ki-67 proliferative index. Combined inhibition of PDGFR and HDAC attenuates proliferation and invasion in chordoma cells deficient for PTEN.

## Introduction

Chordoma is a primary bone cancer believed to arise from the remnants of the notochord, a developmental structure located along the midline that defines the primitive axis of the embryo [1–3]. Consistent with its origin, chordomas are largely restricted to opposing ends of the axial skeleton with the majority found at the sacrum and skull base. Histologically they are characterized by a vacuolated or “physaliphorous,” morphology [4, 5]. Chordomas are slow-growing, low-grade bone cancers characterized by frequent local recurrence. Standard therapy consists of surgical resection, but due to the critical location of the tumor and its invasiveness, complete resection is frequently infeasible. Local control presents a major clinical challenge in the management of chordomas and recurrence eventually leads to the patient’s demise. The median survival after diagnosis is 6–7 years, with 5- and 10-year survival rates of 68% and 40%, respectively [1, 3]. Adjuvant therapy consists of radiation therapy, primarily stereotactic delivery of gamma radiation or proton beam radiotherapy [3, 6, 7]. These delivery methods are preferred due to the high doses required to achieve tumor control, the radiosensitive nature of the adjacent structures, and favorable dose fall off compared to conventional radiotherapy. However, the poor overall prognosis makes the development of effective adjuvant treatment regimens critical to improving patient survival.

Although chordomas are considered to be relatively resistant to chemotherapy, there are case reports of occasional response [8–10]. The only prospectively evaluated pharmacotherapeutic approaches consist of three phase II clinical trials. A topoisomerase I inhibitor, 9-nitro-camptothecin, was investigated in 15 patients and appeared to delay progression of disease [11]. Following the immunohistochemical demonstration that PDGFR is activated in a majority of chordomas, patients with chordoma were treated with imatinib mesylate, a tyrosine kinase inhibitor active against PDGFR, BCR-ABL, and KIT [12, 13]. All participating subjects’ tumor showed phosphorylation of the PDGFR. The investigators noted 1 partial response and 35 subjects with stable disease from a total of 50. More recently, lapatinib was investigated in subjects with advanced EGFR-positive chordoma. Six subjects representing 33% of total demonstrated partial response [14]. These experiences show the potential role of chemotherapy in improving outcome for patients with chordoma, although identification of more effective strategies is needed.

We have previously observed that PDGF is both mitogenic and motogenic for chordoma cells cultured from acutely resected surgical specimens [15]. Based on our observation of *in vitro* intertumoral variation in response to recombinant PDGF protein and PDGFR inhibition and the results of the imatinib clinical trial, we hypothesized that chordomas resistant to PDGFR inhibition may possess downstream activation of the pathway.

## Materials and Methods

### Patients and tumor tissues

The University of Pittsburgh Institutional Review Board approved the study, "Prospective and Retrospective Analysis of Chordoma Tissues and Records for Determination of Growth and Invasive Mechanisms.” Written informed consents were obtained from all subjects prior to surgery. For minors, written consents were obtained from parents/guardians. The process was documented by the investigator and research coordinator. The IRB approved the consenting procedure. All tissues included in the study were clinically confirmed to be chordomas by a pathologist. Tumor specimens were collected prospectively after obtaining informed consent from 23 subjects with chordoma. Resected tumor tissue was immediately placed into sterile DMEM-F12 (GIBCO-Invitrogen, La Jolla, CA) tissue culture medium on ice and transported to the laboratory. Tumors were minced with a scalpel and mechanically dissociated by trituration.

### Cell culture

Chordoma cells were cultured in DMEM/F12 medium supplemented with 2 mM glutamine, 1 mM sodium pyruvate, 100 U/ml penicillin, 100 μg/ml streptomycin (Gibco BRL, Gaithersburg, MD), and 10% heat inactivated fetal bovine serum (Gibco BRL) in a humidified incubator at 37 degrees C. Chordoma cells were identified by the characteristic physaliphorous morphology and expression of brachyury by immunohistochemistry and immunoblot. Cells were passaged upon achieving confluence and used up to 10 passages as long as they retained the physaliphorous morphology.

Chordoma cell lines UCH-1 and UCH-2 were obtained from the Chordoma Foundation. UCH-1 and UCH-2 cells were grown on plates coated with 0.005% collagen (Sigma) in a 4:1 mixture of Iscove’s modified Dulbecco’s medium (Invitrogen) and RPMI-1640 (Sigma) media containing 10% fetal bovine serum, 1 mM L-glutamine, and 26 mM sodium bicarbonate in a 37°C humidified tri-gas incubator. Human glioma lines U87MG and T98G were purchased from American Tissue Type Culture Collection (Manassas, VA, USA) and cultured as previously described [16].

### Hypoxic condition

Cells were incubated in a hypoxic chamber (Forma Scientific, Marietta, OH) with a 93:5:2 mixture of N_2_/CO_2_/O_2_. Deoxygenated media were prepared prior to each experiment by equilibrating with a hypoxic gas mixture containing 93% N_2_, 5% CO_2_, and 2% O_2_ at 37 degrees C.

### Antibodies and inhibitors

Anti-phospho-PI3K, anti-PI3K, anti-laminB antibody, anti-phospho-PDGF-α^T720^, anti-PDGF-α, anti-phospho-PDGF-β^T1009^, and anti-PDGF-β antibodies were purchased from Santa Cruz Biotechnology (Santa Cruz, CA, USA). Anti-HIF-1α antibody was purchased from BD Biosciences (San Jose, CA, USA). Anti-PTEN, anti-phospho-mTor^S2448^, anti-mTOR, anti-phospho-Akt^S473^, anti-phospho-Akt^T308^, anti-Akt, anti-phospho-ERK1/2^T202/T204^, and anti-ERK1/2 antibodies were purchased from Cell Signaling (Beverly, MA, USA). Anti-brachyury antibody was purchased from R&D Systems. Anti-mouse-IgG-HRP and anti-rabbit-IgG-HRP secondary antibodies were purchased from Santa Cruz Biotechnology. The PDGFR inhibitor, 3-Fluoro-N-(6,7-dimethoxy-2,4-dihydroindeno[1,2-c]pyrazol-3-yl)phenylamine, was obtained from EMD Chemicals (Gibbstown, NJ, USA, catalog #521233). A concentration of 1 μM was used for these studies. LBH589 was obtained from Selleck (Houston, TX, USA, catalog #S1030).

### Enzyme-linked immunosorbent assay (ELISA) for detection of vascular endothelial growth factor (VEGF)

Levels of VEGF protein in the media were determined by ELISA using a commercial kit (R&D Systems, Minneapolis, MN, USA) as previously described [16].

### Immunoblot and immunohistochemistry

These were performed as previously described [17]^-^[18]. Briefly, immunoblot analyses were performed by cell lysis in a buffer consisting of 20 mM Tris-HCl (pH 7.4, 150 mM NaCl, 1mM EGTA, 1% Triton X-100, 2.5 mM sodium pyrophosphate, 1 mM β-glycerol phosphate, 1m M Na_3_VO_4_, 1 μM/ml leupeptin and 1mM phenylmethylsulfonyl fluoride). After sonication, lysates were clarified by centrifugation at 12,000 x *g* for 10 minutes at 4°C, and protein content in the supernatant was measured according to the Bradford method. Aliquots (30–50 μg of protein per lane) of total protein were separated by 7.5–15% SDS-polyacrylamide gel electrophoresis and blotted onto nitrocellulose transfer membrane. Each membrane was blocked with 5% non-fat dry milk in TBS-T for 1 hour at room temperature, followed by incubation with the appropriate primary antibodies overnight at 4°C. After extensive washing with TBS-T, each membrane was incubated with horseradish peroxidase-conjugated secondary antibodies at 1:1000 dilution for 1 hour at room temperature in TBS-T. Detection was performed using chemiluminescence reagent. Immunohistochemistry on paraffin-embedded chordoma tumor specimen was performed using antibody against brachyury (R&D Systems). 5 μm sections were deparaffinized and underwent heat-induced epitope retrieval. Slides were then incubated with primary antibody and detected using avidin-biotin-peroxidase complex with 3,3’-diaminobenzidine as the chromogen.

### Brachyury expression analyses

Brachyury was detected using both immunoblot and immunohistochemistry. Nuclear expression was confirmed by immunohistochemistry and by immunoblot performed on isolated nuclear protein fraction.

### Nuclear extraction

We isolated nuclear proteins using a commercially available kit (Pierce Protein Biology Products, Rockford, IL) following the manufacturer’s instructions.

### PCR-based microsatellite LOH analysis

Manual microdissection of the tissue sample was performed and specimens with a minimum of 50% of tumor cells in a microdissection target were accepted for analysis. DNA was isolated using standard laboratory procedures. Optical density readings were obtained. The assay utilized 2 microsatellite markers on chromosome 10q23 near the *PTEN* locus (D10S520, D10S1173). PCR was performed and the PCR products were analyzed using capillary gel electrophoresis on GeneScan ABI 3730 (Foster City, CA). Relative fluorescence was determined for individual alleles and the ratio of peaks was calculated. Neoplastic tissue was then analyzed to detect loss of heterozygosity. As normal tissue was usually not available, peak height ratios falling outside of 2 standard deviations beyond the mean for each polymorphic allele paring were assessed as showing loss of heterozygosity.

### Invasion assay

The BD BioCoat Matrigel Invasion Chamber assay system was used to study the effects of inhibitors on chordoma cell invasion (BD Biosciences, MA). Briefly, precoated filters (8 μm pore-size, Matrigel 100 μg/cm^2^) were rehydrated and seeded with 2.5 x 10^4^ cells in 500 μl of medium with or without inhibitors in triplicates into the upper part of each chamber. The lower compartment was filled with 750 μl of serum-free DMEM/F-12 supplemented with 0.1% BSA. After incubation for 18 h at 37 degrees C, non-invaded cells on the upper surface of the filter were wiped with a cotton swab, and migrated cells on the lower surface of the filter were fixed and stained with Diff-Quick kit. Invasiveness was determined by counting cells in five microscopic fields per well, and the extent of invasion was expressed as an average number of cells per microscopic field.

### Cell proliferation assay

3-(4,5-dimethylthiazol-2-yl)-5-(3-carboxymethoxyphenyl)-2-(4-sulfophenyl)-2H-tetrazolium (MTS) assay was used according to the manufacturer’s recommendations (Promega, Madison, WI). UCH-1 and C-18 chordoma cells were infected with PTEN-encoding adenoviruses (Ad-PTEN) or control adenoviruses (Ad-con) at an MOI (multiplicity of infection) = 10. After 24 h, the cells were treated with the PDGFR inhibitor or DMSO control. The cells were subsequently collected every day for five days and counted with a hemocytometer.

### Annexin V-PE and 7-AAD flow cytometry

The effects of PTEN expression on PDGFR inhibitor-mediated effects on apoptosis were assessed by Annexin V-PE and 7-AAD flow cytometry. UCH-1 and C18 cells were infected with Ad-PTEN or Ad-con for 24 h and then were treated with PDGFR inhibitor for 48 h. Cells were harvested and stained with Annexin V-PE and 7AAD according to the manufacturer's instructions. Cell samples were analyzed on a FACsan flow cytometer and apoptotic fractions were determined.

### Vectors and Transfections

Adenoviruses encoding wild-type PTEN (Ad-PTEN) and control adenovirus (Ad-control) were constructed as described previously [19, 20]. Cells were infected with adenovirus vectors (MOI = 10) for 24 h before treatment with PDGFR inhibitor.

### Statistical analysis

Statistical analysis was carried out using Graphpad InStat 3 software (GraphPad Software, Inc., San Diego, CA, USA). Results were considered statistically significant at *p* < 0.05.

## Results

### Patient characteristics

Chordoma tissues from 23 patients were analyzed (Table 1). There were 18 men and 5 women. Ages ranged from 6 to 77 years. Seventeen were cases at first presentation and 6 were recurrent tumors. All specimens showed nuclear expression of brachyury by immunohistochemistry. We show one representative immunohistochemical staining and four immunoblots with control cancer cell lines that do not express brachyury (S1 Fig). Immunoblot performed on isolated nuclear protein fraction indicates nuclear localization of brachyury (S6 Fig).

**Table 1:** Patient and tumor characteristics from 23 consecutive surgically resected chordomas.

Case	Sex	Age (Years)	Site	Type	10q LOH	Ki-67 (%)
1	M	22	Clivus	Primary	No	2
2	M	35	Clivus	Primary	No	1.5
3	M	61	Clivus	Primary	No	4
4	M	70	Clivus	Primary	No	0.5
5	M	63	Clivus	Primary	Yes	4
6	M	32	Clivus	Recurrent	No	2.5
7	F	77	Cervical	Primary	No	15
8	M	30	Clivus	Primary	No	2.5
9	F	37	Clivus	Recurrent	No	0.5
10	M	20	Clivus	Primary	No	1.5
11	F	72	Clivus	Primary	No	5
12	F	46	Clivus	Recurrent	No	2.5
13	M	28	Clivus	Primary	Yes	20
14	M	41	Clivus	Primary	Yes	10
15	M	52	Clivus	Primary	No	5
16	M	26	Clivus	Primary	Yes	3
17	M	66	Clivus	Recurrent	No	5
18	M	6	Clivus	Recurrent	Yes	12.5
19	M	60	Clivus	Primary	No	0.5
20	M	43	Clivus	Primary	No	1
21	M	73	Clivus	Primary	Yes	10
22	F	24	Clivus	Primary	No	0.5
23	M	56	Clivus	Recurrent	No	12.5

Abbreviations: LOH, Loss of heterozygosity.

### Loss of PTEN

**Fig 1:**
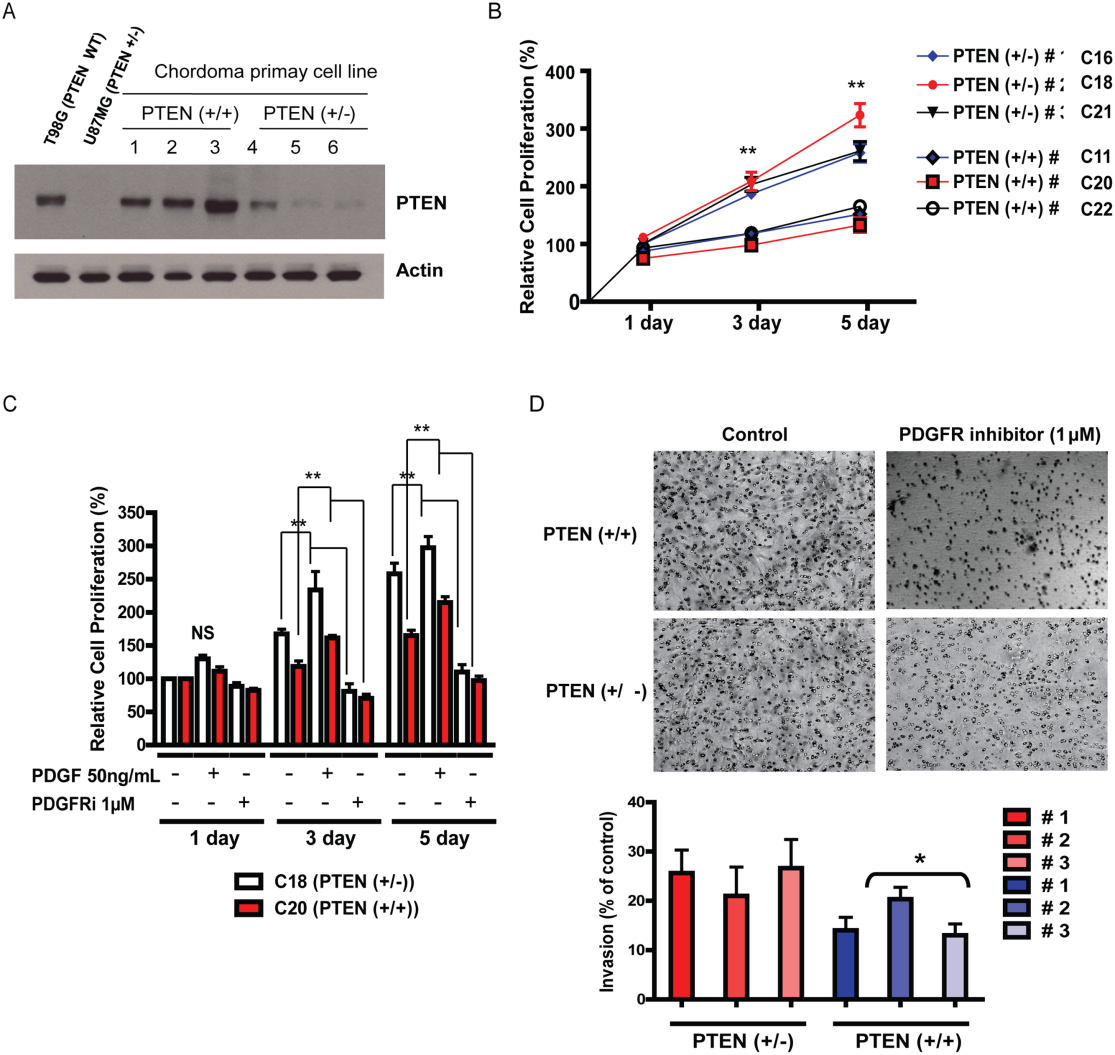
*PTEN* disruption in Chordoma is associated with enhanced *in vitro* proliferation and invasion. (A) A representative immunoblot for PTEN expression demonstrating a decreased level of expression of PTEN in the chordoma specimens with LOH at 10q23 locus compared with wild type chordomas. T98G is used as a positive control and U87MG is used as a negative control for PTEN expression. This is a representative blot from three independent experiments. (B)Cell proliferation assay assessed by the MTS method demonstrating a higher rate of proliferation among chordoma cell lines with *PTEN* LOH compared with wild type. ** P<0.05, Two sample independent student’s t test. (C and D) Cell proliferation assay assessed by MTS method demonstrating a higher rate of proliferation for chordoma lines with *PTEN* LOH compared with wild type. Addition of 50ng/mL of PDGF results in increased proliferation in both *PTEN* LOH and wild type cells. Addition of 1 uM of PDGF inhibitor resulted in decreased proliferation compared to untreated control. **P<0.05, Two sample independent student’s t test compared to untreated control from respective cell lines. (E) A representative matrigel invasion assay from demonstrating significant decrease in invasion in response to PDGF inhibition among *PTEN* wild type chordoma lines compared with *PTEN* +/- cells. These images are representative of 3 independent experiments. *P<0.05, Two sample independent student’s t test.

PCR-based microsatellite LOH analysis of the 10q23 locus revealed LOH at 10q23, indicating loss of PTEN gene in 6 of 23 specimens (S2 Fig). In agreement, immunoblot of the primary culture cells established from the chordoma tissues with LOH at 10q23 showed decreased or absent PTEN expression (Fig 1). Chordoma tissues without LOH at 10q23 demonstrated PTEN protein expression (Fig 1). Previously characterized cell lines served as positive and negative controls (Fig 1).

### Chordoma cells deficient for PTEN show increased in vitro growth and resistance to PDGFR inhibition

To characterize the role of PTEN on growth of chordoma cells, primary cultures were established from 20 of 23 tumor specimens. Ten specimens (all PTEN wild type) showed very slow growth rates, limiting experimental use. Therefore, primary cultures were established from 14 PTEN wild type and 6 PTEN deficient tumor specimens. From the remaining 4 PTEN wild type chordomas, three demonstrated particularly robust *in vitro* growth. These PTEN wild type tumors were used to compare growth rate and invasiveness to the PTEN deficient chordoma tumor cells. Chordoma cells lacking PTEN expression demonstrated accelerated *in vitro* growth rate with shorter doubling time compared to PTEN wild type chordomas as determined by cell number and by MTS proliferation assay (Fig 1). To determine the effect of PTEN status on chordoma cellular response to PDGFR inhibition, chordoma cells from PTEN deficient and wild type tumors were exposed to a PDGF receptor tyrosine kinase inhibitor. We investigated the effect of PDGFR inhibition on proliferative rate and invasive capacity. We did not detect a significant difference in proliferative rate in response to the PDGFR inhibitor (Fig 1). Also, the *in vitro* invasive capacity of PTEN wild type and PTEN deficient tumors were similar (Fig 1). However, PTEN deficient tumors showed greater resistance to PDGFR inhibition on invasion compared to PTEN wild type cells (Fig 1).

### PTEN restoration results in attenuation of growth

**Fig 2:**
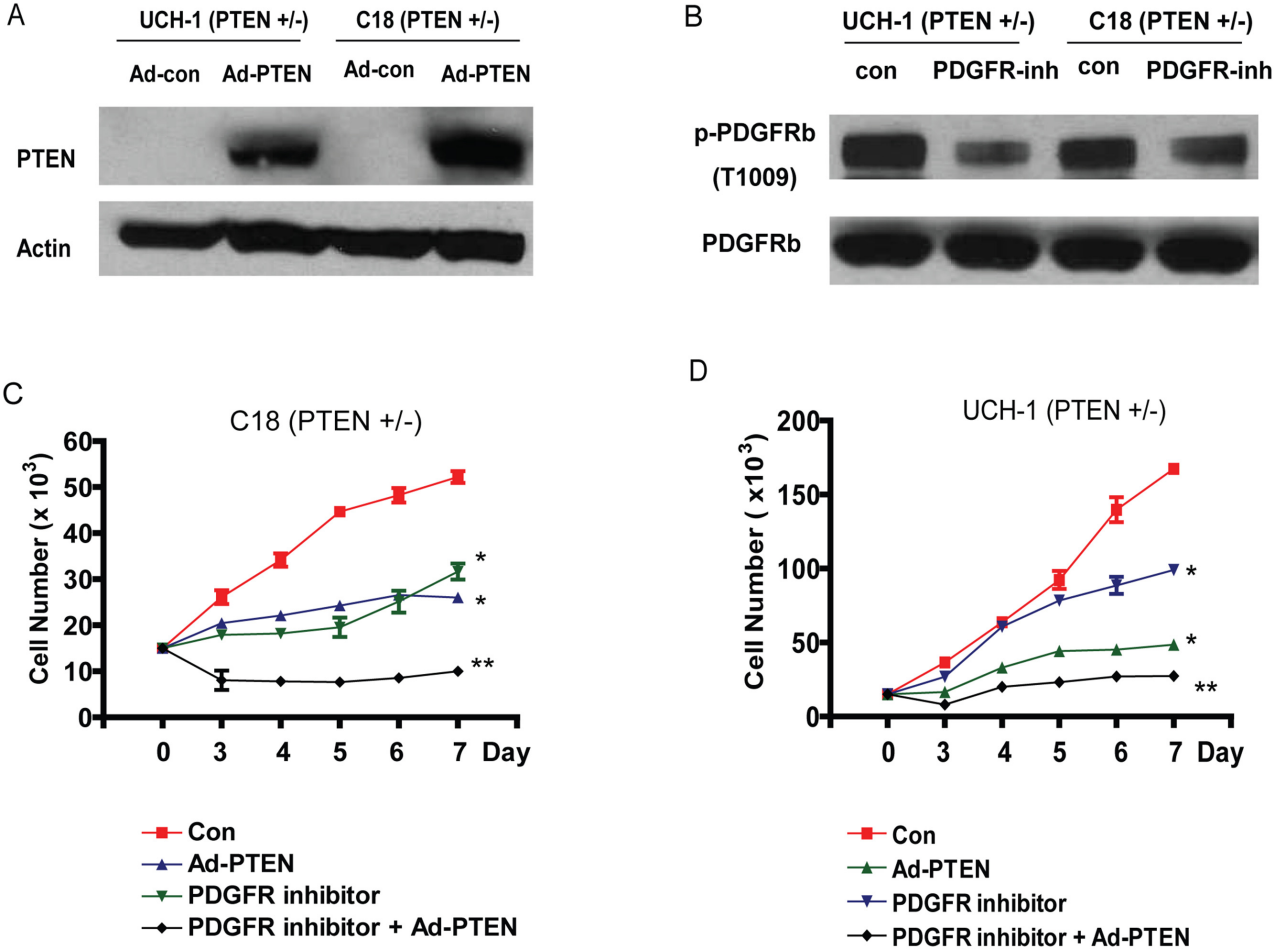
Restoration of PTEN expression attenuates *in vitro* proliferation of Chordoma cells. (A) A representative immunoblot demonstrating reconstitution of PTEN expression via adenoviral delivery. (B) A representative immunoblot for phospho-PDGFRb demonstrating decreased expression with administration of PDGFR inhibitor compared with vehicle controls. (C and D) Cell proliferation assays assessed by the MTS method for C18 cell line (*PTEN* +/-) and UCH1 cell line (*PTEN* +/-), respectively, demonstrating decreased proliferation with restoration of *PTEN* and administration of PDGFR inhibitor. *P<0.05, Two sample independent student’s t test compared with untreated control. **P<0.05, Two sample independent student’s t test compared with PDGFR inhibitor group.

**Fig 3:**
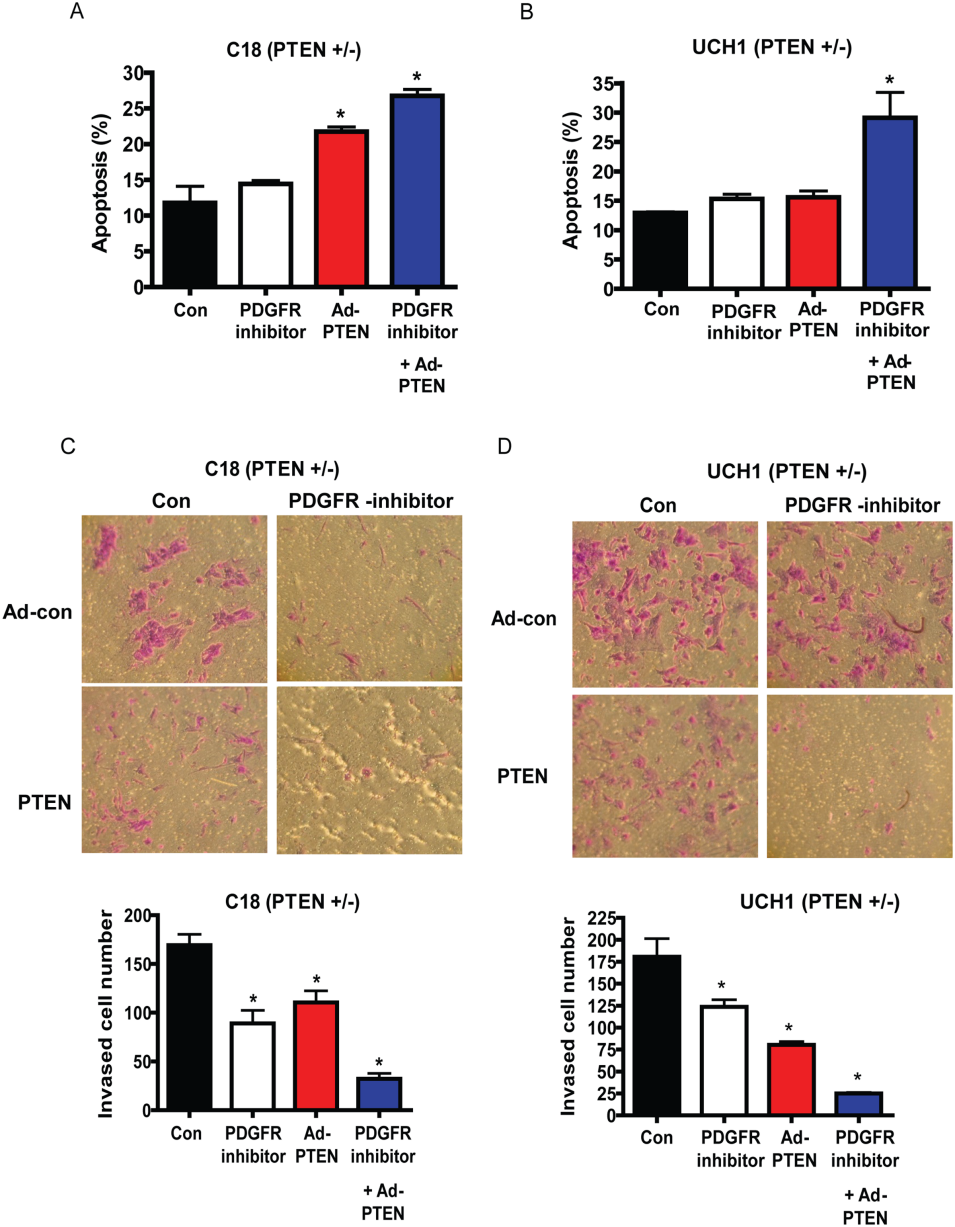
PTEN reconstitution enhances Chordoma cell sensitivity PDGFR inhibition . (A and B) Representative apoptosis assays for (A) C18 cell line (*PTEN* +/-) and (B) UCH1 cell line (*PTEN* +/-), respectively, assessed by Annexin-V and 7-AAD florescence demonstrating significantly higher rates of apoptosis with restoration of *PTEN* and administration of PDGFR inhibitor compared with control. *P<0.05, Two sample independent student’s t test. This is representative of three independent experiments. (C and D) Representative matrigel invasion assays with the C18 cell line (*PTEN* +/-) and UCH1 cell line (*PTEN* +/-), respectively, demonstrating significant decrease in invasion in response to PDGF inhibition with restoration of *PTEN* expression. These images are representative of 3 independent experiments. *P<0.05, Two sample independent student’s t test.

To confirm that PTEN deficiency drives chordoma proliferation and resistance to PDGFR inhibition and is not merely a bystander, we restored PTEN expression in two chordoma cell lines lacking PTEN. Lines C18, C21 (primary culture) and UCH-1 (established chordoma cell line) showed attenuation of proliferative rate (Fig 2 and S5 Fig). Increase in apoptosis and attenuation of invasive capacity was only seen in the primary culture C18 and not with UCH-1, an established cell line (Fig 3). However, PTEN restoration established sensitivity to PDGR inhibition in both cell lines (Fig 3 and S2 Fig).

### HDAC inhibition attenuates expression of the hypoxia inducible factor 1 alpha (HIF-1α) and results in decreased chordoma cell proliferation and invasion

**Fig 4:**
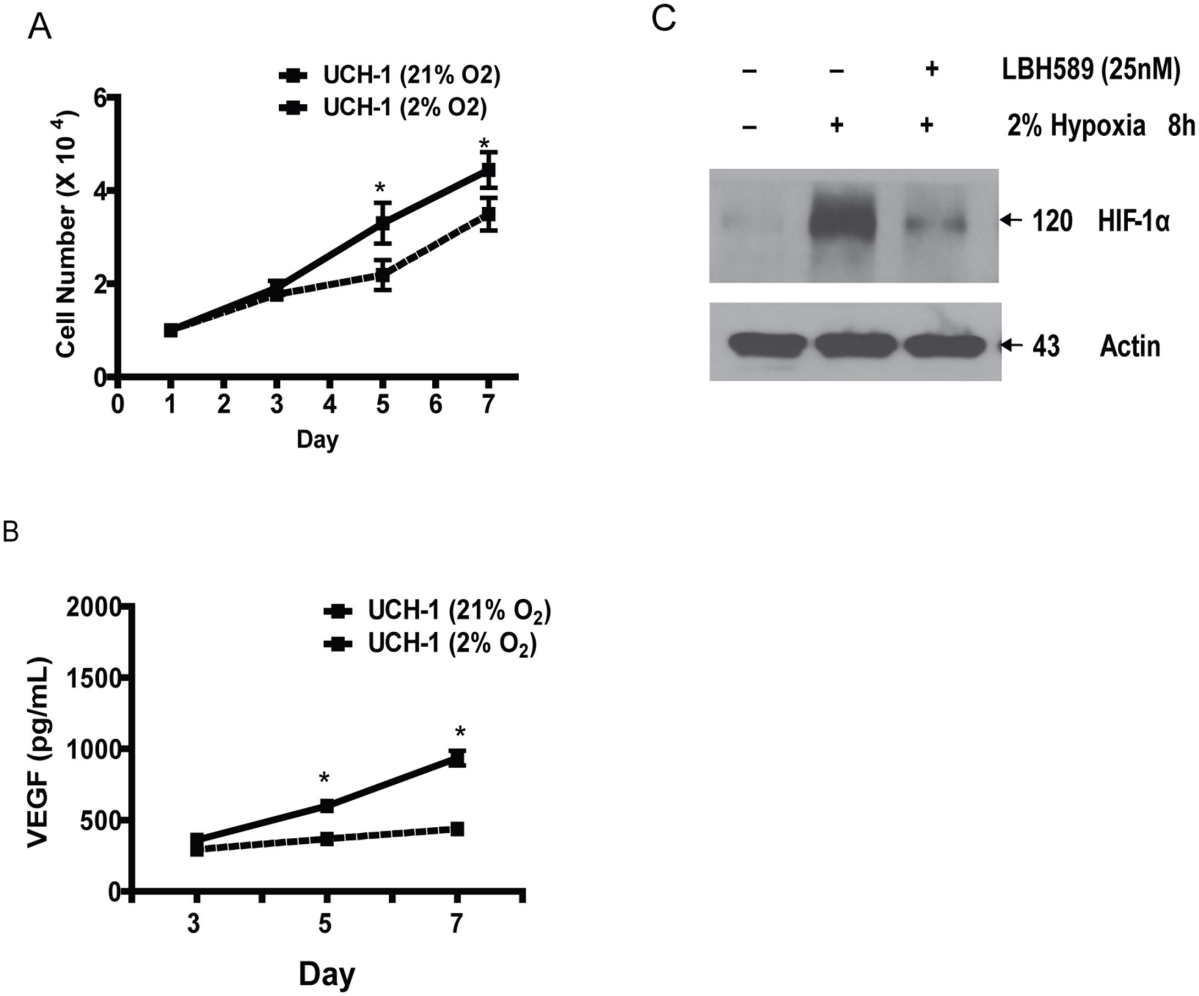
Hypoxic expansion of Chordoma cells is mediated by stabilization of HIF-1α. (A) A representative cell proliferation assay assessed by the MTS method for the UCH1 cell line (*PTEN* +/-) demonstrating increased proliferation with exposure to hypoxia (2% Oxygen) compared to normoxia (21% Oxygen). This is representative of 3 independent experiments. *P<0.05, Two sample independent student’s t test. (B) A representative VEGF expression assay as assessed by ELISA from cell culture supernatants for the UCH1 cell line (*PTEN* +/-) demonstrating increased expression of VEGF with exposure to hypoxia compared with normoxic controls. This is representative of 3 independent experiments. *P<0.05, Two sample independent student’s t test. (C) A representative immunoblot for HIF-1α demonstrating increased expression of HIF-1α with hypoxia exposure compared with normoxia and abrogation of this increase with administration of HDAC inhibitor.

Chordomas harbor regions of intratumoral hypoxia that can contribute to treatment resistance.[21, 22] To identify potential therapeutics effective in low oxygen setting, we characterized *in vitro* propagation of chordoma cells at 2% oxygen and observed increased proliferative rate (Fig 4) and level of vascular endothelial growth factor (VEGF), a transcriptional target of HIF-1α (Fig 4). Treatment with HDAC inhibitor LBH589 (25 nM) led to reduction of HIF-1α level (Fig 4).

### Combined PDGFR and HDAC inhibition is associated with reduction in invasion and proliferation

**Fig 5:**
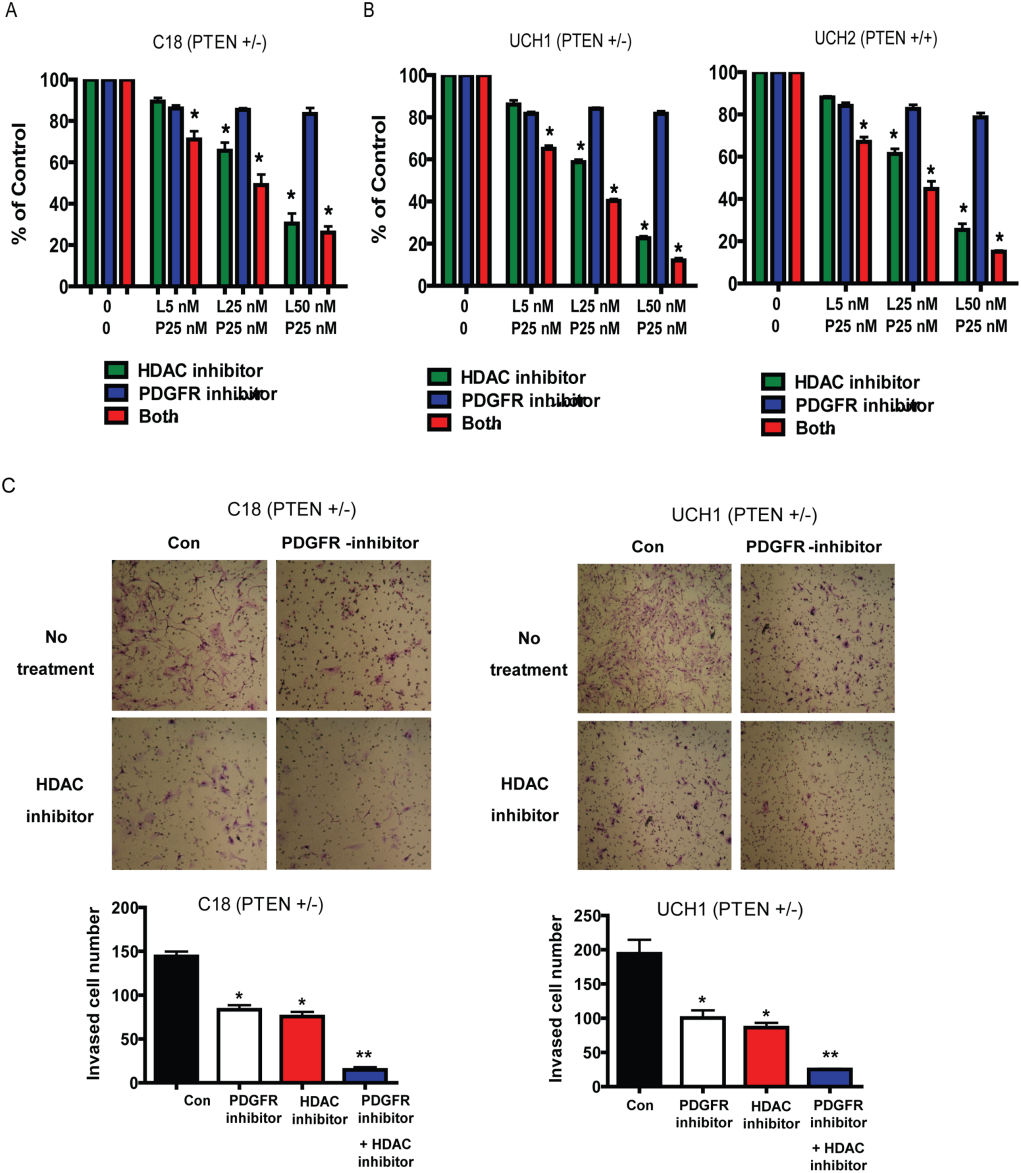
Combined PDGFR and HDAC inhibition strikingly reduces proliferation and *in vitro* invasion of Chordoma cells. (A) Cell proliferation assay assessed by MTS method using C18 (*PTEN* +/-) cells demonstrating significantly decreased proliferation in response to HDAC inhibition and combined treatment with a PDGFR inhibitor compared to controls. This is representative of 3 independent of experiments. *P<0.05, Two sample independent student’s t test. (B) Cell proliferation assays assessed by MTS method using UCH1 (PTEN +/-) and UCH2 (PTEN +/-) cells demonstrating significantly decreased proliferation in response to HDAC inhibition and combined treatment with a PDGFR inhibitor compared to controls. This is representative of 3 independent of experiments. *P<0.05, Two sample independent student’s t test. (C) Representative matrigel invasion assays with the C18 cell line (*PTEN* +/-) and UCH1 cell line (*PTEN* +/-), respectively, demonstrating significant decrease in invasion in response to PDGFR inhibition, HDAC inhibition and PDGFR and HDAC inhibition together. These images are representative of 3 independent experiments. *P<0.05, Two sample independent student’s t test compared to untreated control. **P<0.05, Two sample independent student’s t test compared to PDGFR inhibitor alone.

Chordoma cells with intact PTEN expression and those deficient for PTEN both demonstrate greater potential therapeutic response to the combination of PDGFR inhibitor and HDAC inhibition (Fig 5). The benefit of combined inhibition was particularly significant for attenuation of invasion (Fig 5).

## Discussion

Identifying the determinants of tumor resistance to therapy is important for improving patient care and will likely lead to better understanding of the underlying biology [23]. In this study, we profiled 23 chordoma specimens, and investigated mechanisms of *in vitro* proliferation and invasion of chordoma cells to identify effective combinatorial regimens. Because availability of established chordoma cell lines is limited, we relied on primary culture specimens. We found that primary culture of chordoma can be established, and PTEN is lost in a subset of chordoma [24]. This loss leads to enhanced *in vitro* growth and relative resistance to PDGFR inhibition compared to chordomas with intact PTEN expression. Interestingly, we observed that PTEN loss was associated with greater resistance to proliferation compared to invasion. Such observation may in part explain paucity of tumor shrinkage, but high number of subjects with stable disease with the use of single agent imatinib for chordoma [13]. Determining the functional significance of PTEN loss in chordoma and the observation that such loss may result in resistance to upstream inhibition may provide design of therapeutic strategies to overcome such disruptions.

This study was performed because: 1) experience from a clinical trial demonstrated variable clinical response of chordomas to PDGFR inhibition despite all tumors showing activation of PDGFR; and 2) our *in vitro* laboratory observation of intertumoral variation in growth rate and response to PDGR inhibition of chordoma cells. To identify the potential molecular basis for such observations, we profiled chordoma specimens and investigated the *in vitro* growth characteristics. All histologically confirmed chordoma specimens demonstrated nuclear expression of brachyury. Because brachyury expression becomes restricted to the notochord during development, this marker is useful in distinguishing chordomas from other tumors [25–27].

Addition of recombinant PDGF to the chordoma cells led to downstream activation of appropriate signaling pathways (S3 Fig). We observed LOH at 10q23, a locus that contains the *PTEN* gene, in a subset of chordomas. LOH at 10q23 was statistically significant for increased Ki-67, an established negative prognostic indicator for chordoma (S4 Fig). *PTEN* gene encodes a phosphatidylinositol-3,4,5-triphosphate (PIP3) phosphatase that functions as an important negative regulator of the PI3K-Akt signaling pathway. Considering that the PI3K-Akt network activity is amplified in many human cancers, it is not a surprise that PTEN is one of the most commonly disrupted tumor suppressors in cancers [28]. Investigations into the pattern of *PTEN* loss in cancers show that LOH is a significantly more common finding than biallelic gene inactivation [29]. Subsequent studies showed that the loss of heterozygosity at the *PTEN* locus is important in tumorigenesis because PTEN is a haploinsufficient tumor suppressor [29–31].

In half of the chordoma cases with LOH at 10q23, we observed absence of PTEN protein expression. There are several possible explanations for loss of PTEN protein with deletion of one allele. Mutation of the *PTEN* promoter on the retained allele can result in gene silencing [32]. Also epigenetic mechanisms, specifically methylation of the promoter have been described [33–36].

It has been previously demonstrated that PTEN deficient cells exhibit increased proliferation, reduced apoptosis, and enhanced migration [37–39]. Chordoma cells lacking PTEN protein expression indeed showed increased *in vitro* growth rate and shorter doubling time compared with PTEN wild type chordoma cells. PTEN loss was associated with greater resistance to PDGFR inhibition on invasion-migration assay in comparison to PTEN wild type chordoma. Such observations are intriguing because chordomas are not rapidly growing tumors and clinical progression frequently involves slow but insidious invasion into surrounding structures. Another potentially important clinical consideration resulting from PTEN deficiency concerns genetic instability. Loss of PTEN has been linked to genetic instability of cancers through a mechanism of CHK1 phosphorylation from unopposed activation of AKT [40]. This leads to accumulation of DNA double-strand breaks due to cytoplasmic sequestration of CHK1 [41]. In agreement with experimental findings, increased aneuploidy is observed in human cancers with low PTEN expression [40, 42, 43]. It will be interesting to see if chordomas with PTEN loss demonstrate greater genetic instability. Also, as expected from inhibition of CHK1, PTEN deficient cells show impaired DNA repair checkpoint regulations in response to ionizing radiation [40]. Because chordoma patients are often treated with external beam radiation therapy after surgery, there is a theoretical risk for tumors that have PTEN loss to accelerate genetic instability upon exposure to radiation.

To further investigate the functional significance of PTEN loss in chordomas, we restored its expression in *PTEN* disrupted tumors using an adenoviral vector. Reintroduction of PTEN was associated with reduction in proliferation and enhanced therapeutic sensitivity to PDGFR inhibitor. However, in vivo reconstitution of PTEN is technically inefficient at this time. Due to this technical limitation, we sought to identify another target that could act in concert with PDGFR inhibition. Considering the consequences of PTEN loss, inhibitors of Akt and mTOR were investigated serially in combination with PDGFR inhibitor. Neither combination was compelling. mTOR inhibition merely resulted in reduction in the size of the chordoma cells and had little effect on proliferation and invasion (Data not shown).

We chose to move forward with the combination of PDGFR and HDAC inhibition because: 1) HDAC inhibition has shown to attenuate cell migration, and chordomas for the most part progress by local invasion; 2) Chordomas are characterized by regions of intratumoral hypoxia and HDAC inhibition can down regulate expression of HIF-1α, a master regulator of the hypoxic response [21]; 3) Receptor tyrosine kinase signaling pathway overlaps with hypoxic signaling cascade and PTEN loss is associated with enhance HIF-1α expression [44]; 4) HDAC inhibitor mediated cytotoxic mechanism is distinct from tyrosine kinase inhibition. We observed that chordoma cells propagated at low oxygen tension had a proliferative advantage and expressed both HIF-1α and its downstream target, VEGF. HDAC inhibition led to reduction in chordoma cell proliferation and invasion, and HIF-1α expression. A recent report describing cytotoxic activity of HDAC inhibitors in chordoma further support our findings [45]. The combination of PDGFR and HDAC inhibitors demonstrated striking reduction in chordoma cell invasion, irrespective of PTEN status.

Our report illustrates the presence of therapeutically relevant genetic heterogeneity in chordoma. Intertumoral histologic heterogeneity is well recognized with descriptions of classic, chondroid, and undifferentiated types. However, because most patients had skull base tumors, it will be important to confirm our findings in sacral chordomas. This is particularly important because the regional developmental pattern of notochord shows contributions from distinctive cell types [46]. Nevertheless, within the chordomas of clival origin we found molecular heterogeneity that may have clinical relevance and allow for stratification of patients for therapy. The observation that PTEN loss grants a greater resistance to PDGFR inhibition on migration-invasion rather than growth was surprising but is consistent with the biological characteristics of the disease. Characterization of tumors to identify key loss and gain of functions will not only allow for selection of rational agents, but equally important is to define a more relevant endpoint to determine outcome. Future clinical studies of imatinib in chordoma may benefit from a PTEN subgroup analysis to determine rate of progression with less emphasis on response. Finally, our findings provide compelling preclinical rationale for a biomarker-driven clinical study of combined inhibition of PDGFR and HDAC in chordomas.

## Supporting Information

S1 Fig Characterization of Chordoma cells. (A) Hematoxylin and eosin staining of a surgical specimen from our series demonstrating the typical physaliphorous morphology of chordomas. (B) Immunohistochemical staining for brachyury of a surgical specimen from our series demonstrating nuclear staining consistent with tissue of notochord origin. (C) Bright field image of chordoma cells cultured from a surgical specimen demonstrating a recapitulation of the physaliphorous morphology. (D) Immunoblot for brachyury demonstrating strong expression of brachyury among the primary chordoma cell lines compared to glioma cell lines (negative controls). This blot is representative of three independent experiments.
(TIF)

S2 Fig Loss of heterozygosity (LOH) of *PTEN*. (A) PCR-based microsatellite LOH analysis of 10q23 locus, region that contains *PTEN*, from a surgical chordoma specimen demonstrating a single peak consistent with LOH at this site. (B)PCR demonstrates two distinct peaks indicating an intact 10q23 locus.
(TIF)

S3 Fig PDGRFR signaling cascade in Chordoma cells. (A) Immunoblot for multiple members of the PDGFR signaling pathway in C18 cells demonstrats activation of signaling within minutes of administration of exogenous PDGF (50 ng/mL). (B) Immunoblot of phosphor-PDGFR in C11, C16, C21 and C22 in respond to PDGF stimulation.
(TIF)

S4 Fig Ki-67 index and PTEN status. This graph demonstrates significantly lower Ki-67 proliferation index among PTEN intact tumors compared with tumors with LOH.
(TIF)

S5 Fig PTEN and association with proliferation. Additional studies performed on PTEN deficient C21 primary culture chordoma cell shows restoration of PTEN retards proliferation and establishes synergy with PDGFR inhibition.
(TIF)

S6 Fig Nuclear expression of brachyury. Immunoblot was performed on isolated nuclear protein fraction to demonstrate nuclear localization of brachyury that further supports immunohistochemical staining.
(TIF)
