# Supplementary figures and images for: Combined PDGFR and HDAC Inhibition Overcomes PTEN Disruption in Chordoma
Source: PLoS One. 2015 Aug 6;10(8):e0134426. doi: 10.1371/journal.pone.0134426 (PMC4527706; doi:10.1371/journal.pone.0134426)

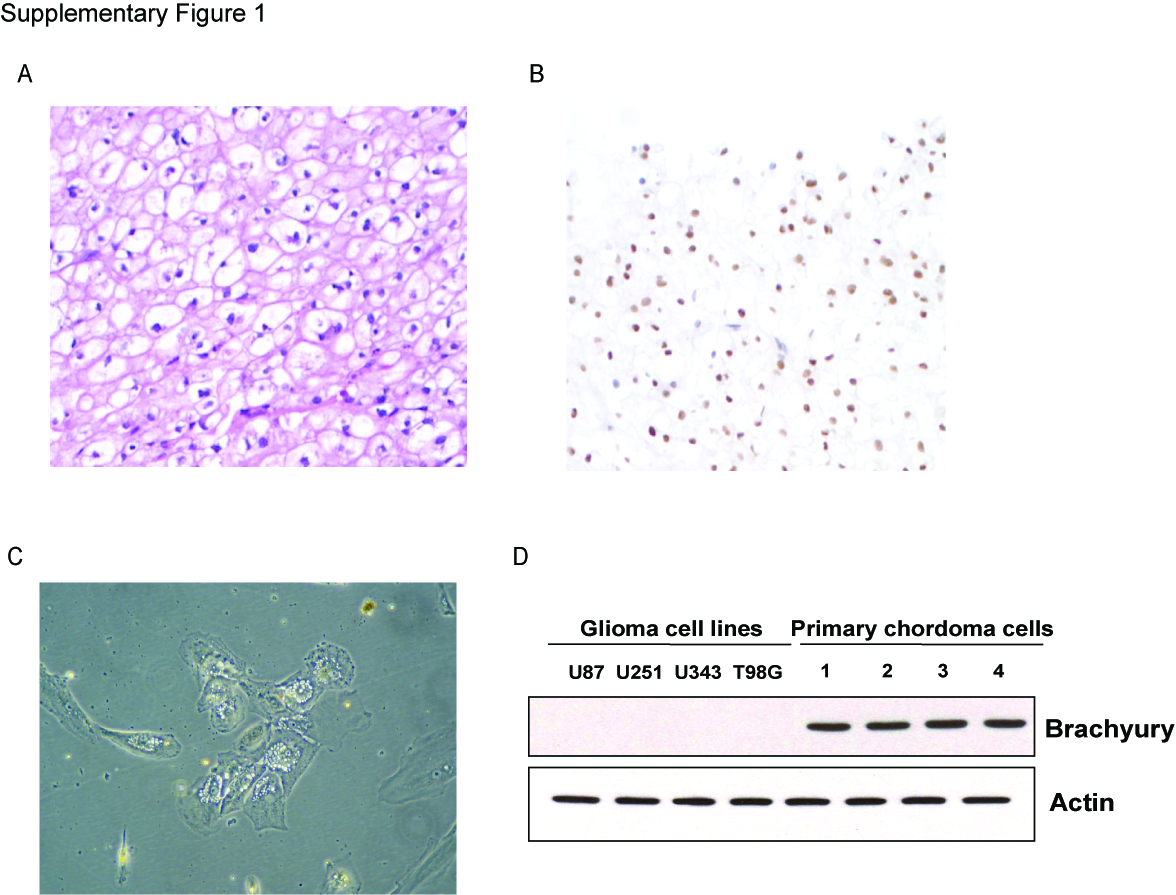

Supplement: S1 Fig — (A) Hematoxylin and eosin staining of a surgical specimen from our series demonstrating the typical physaliphorous morphology of chordomas. (B) Immunohistochemical staining for brachyury of a surgical specimen from our series demonstrating nuclear staining consistent with tissue of notochord origin. (C) Bright field image of chordoma cells cultured from a surgical specimen demonstrating a recapitulation of the physaliphorous morphology. (D) Immunoblot for brachyury demonstrating strong expression of brachyury among the primary chordoma cell lines compared to glioma cell lines (negative controls). This blot is representative of three independent experiments. (TIF) [file pone.0134426.s001.tif]

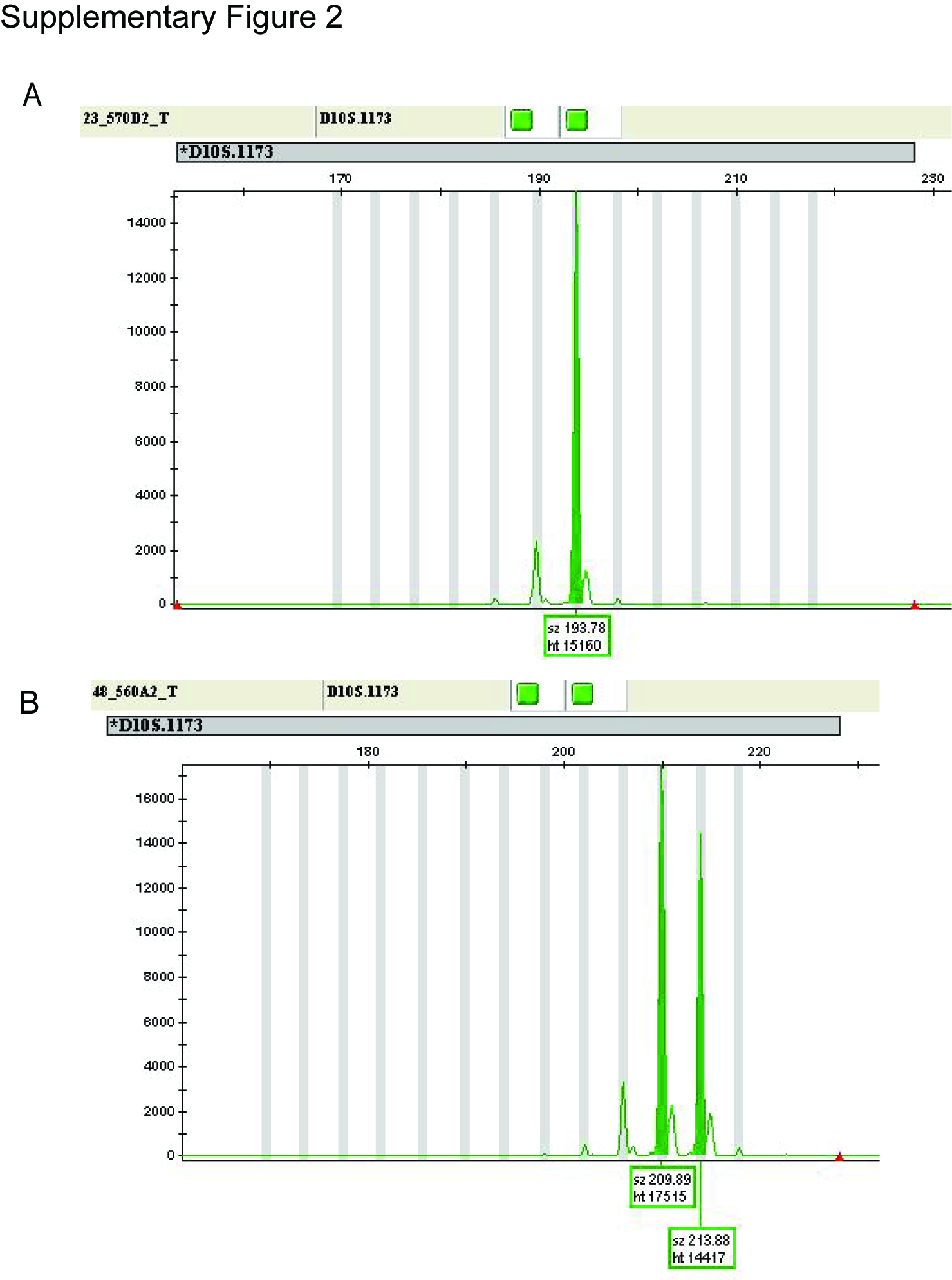

Supplement: S2 Fig — (A) PCR-based microsatellite LOH analysis of 10q23 locus, region that contains PTEN, from a surgical chordoma specimen demonstrating a single peak consistent with LOH at this site. (B)PCR demonstrates two distinct peaks indicating an intact 10q23 locus. (TIF) [file pone.0134426.s002.tif]

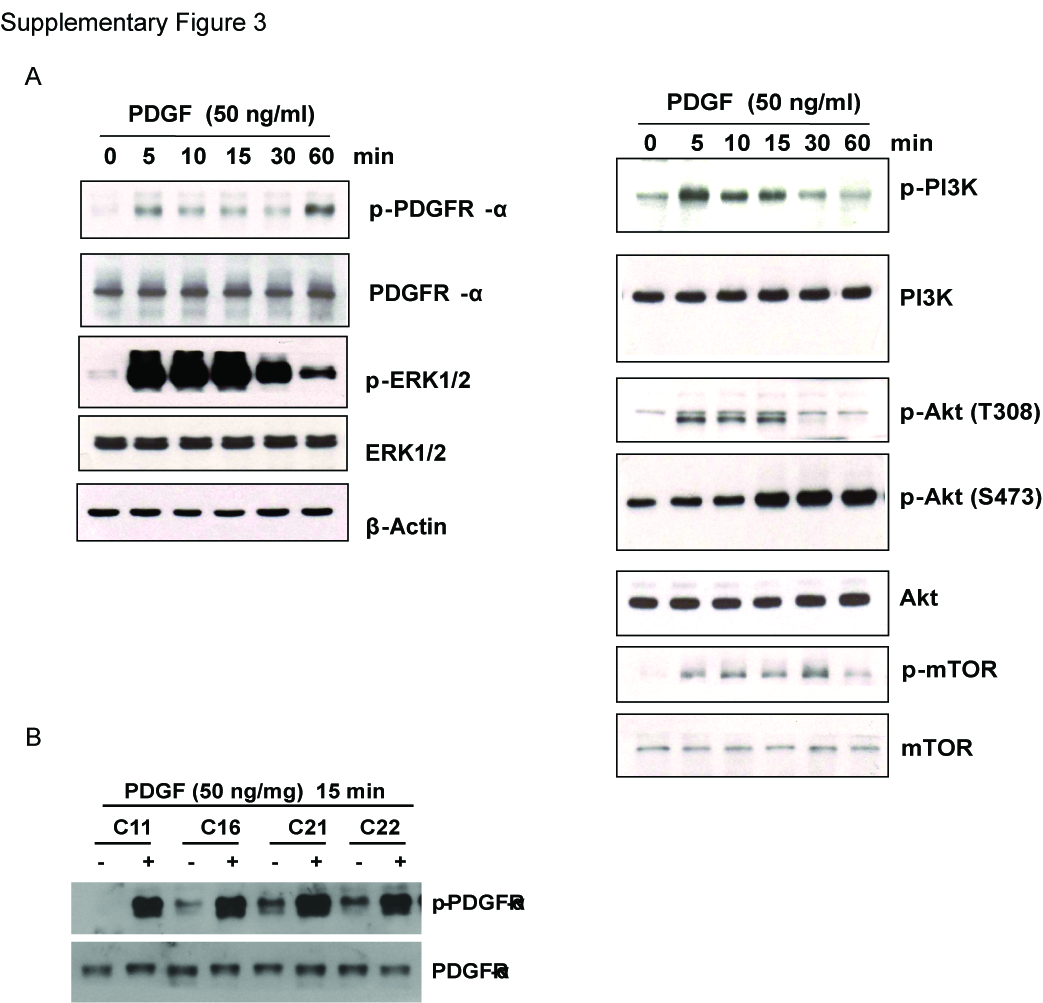

Supplement: S3 Fig — (A) Immunoblot for multiple members of the PDGFR signaling pathway in C18 cells demonstrats activation of signaling within minutes of administration of exogenous PDGF (50 ng/mL). (B) Immunoblot of phosphor-PDGFR in C11, C16, C21 and C22 in respond to PDGF stimulation. (TIF) [file pone.0134426.s003.tif]

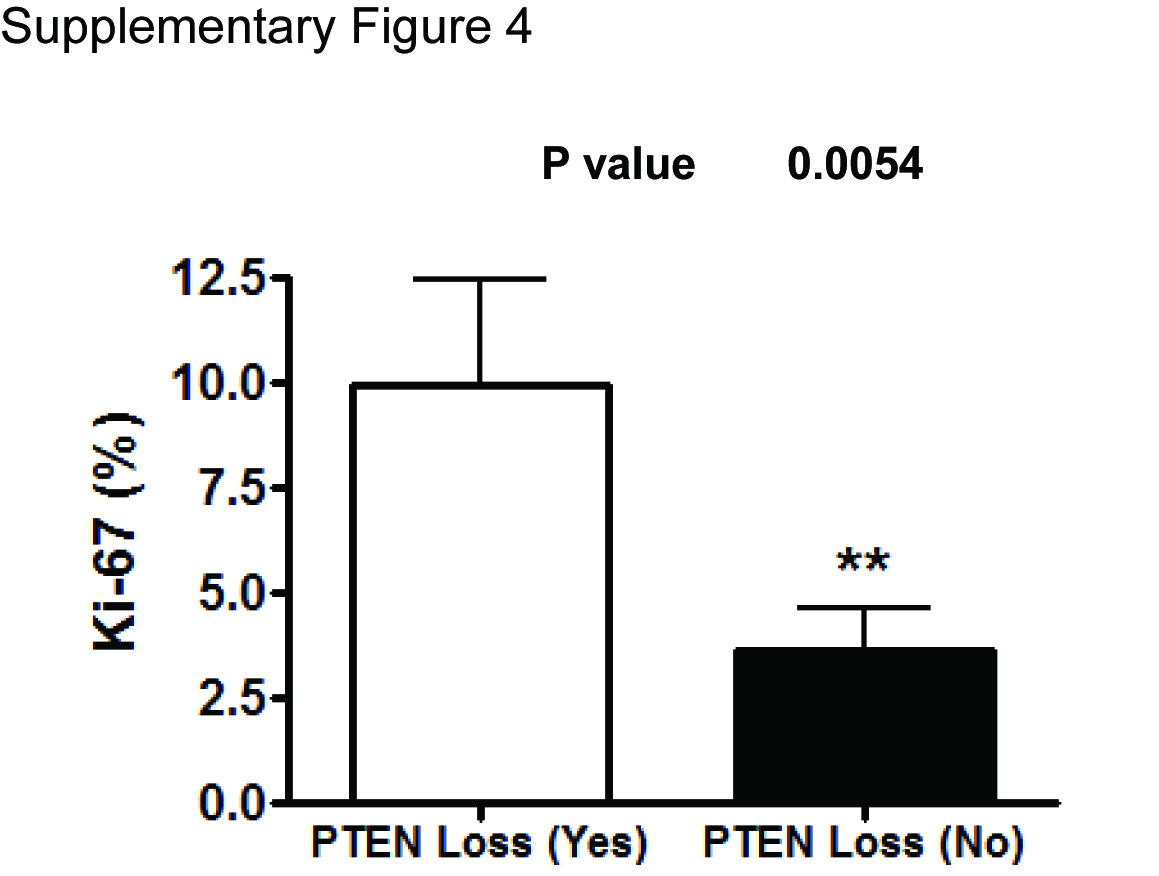

Supplement: S4 Fig — This graph demonstrates significantly lower Ki-67 proliferation index among PTEN intact tumors compared with tumors with LOH. (TIF) [file pone.0134426.s004.tif]

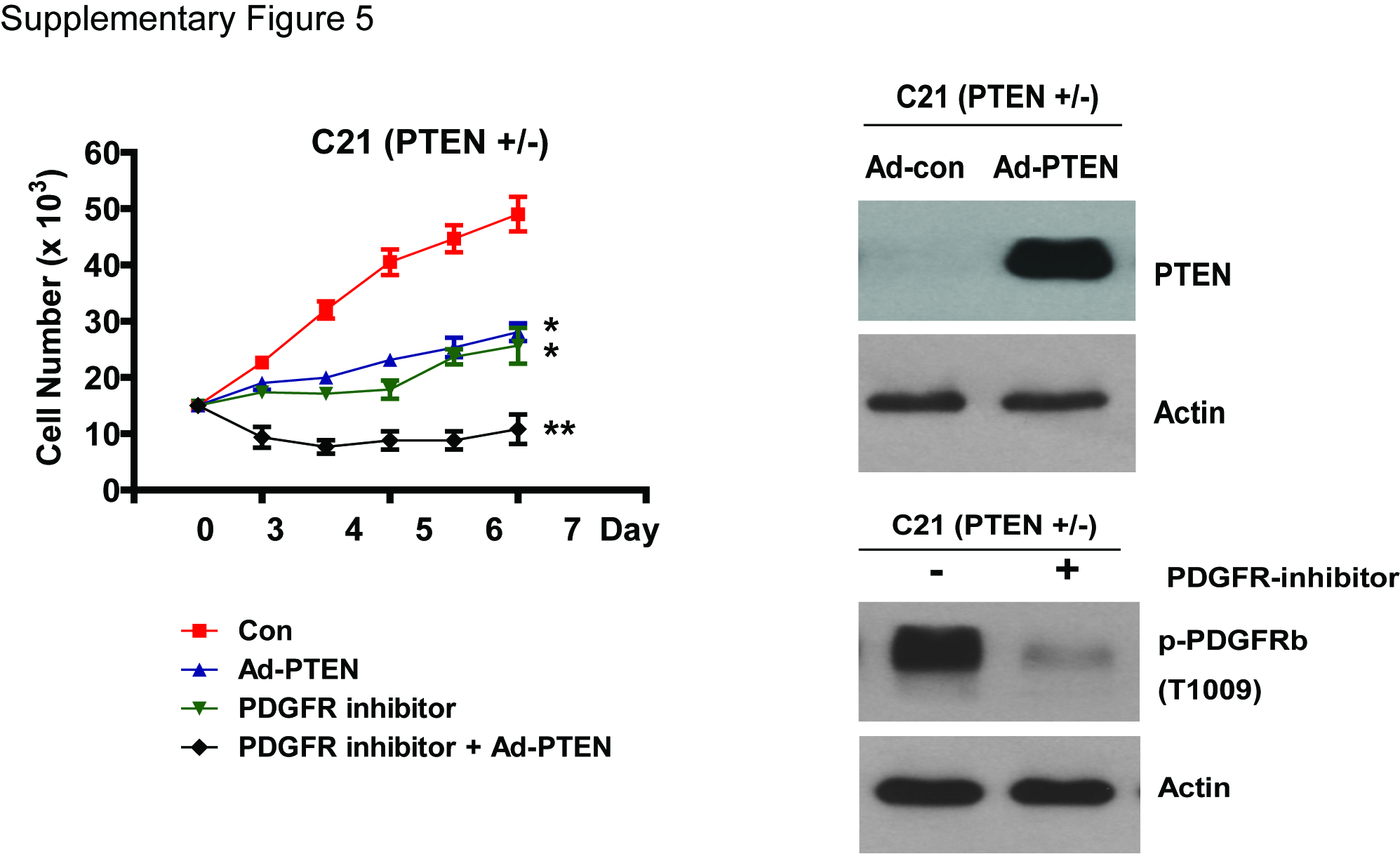

Supplement: S5 Fig — Additional studies performed on PTEN deficient C21 primary culture chordoma cell shows restoration of PTEN retards proliferation and establishes synergy with PDGFR inhibition. (TIF) [file pone.0134426.s005.tif]

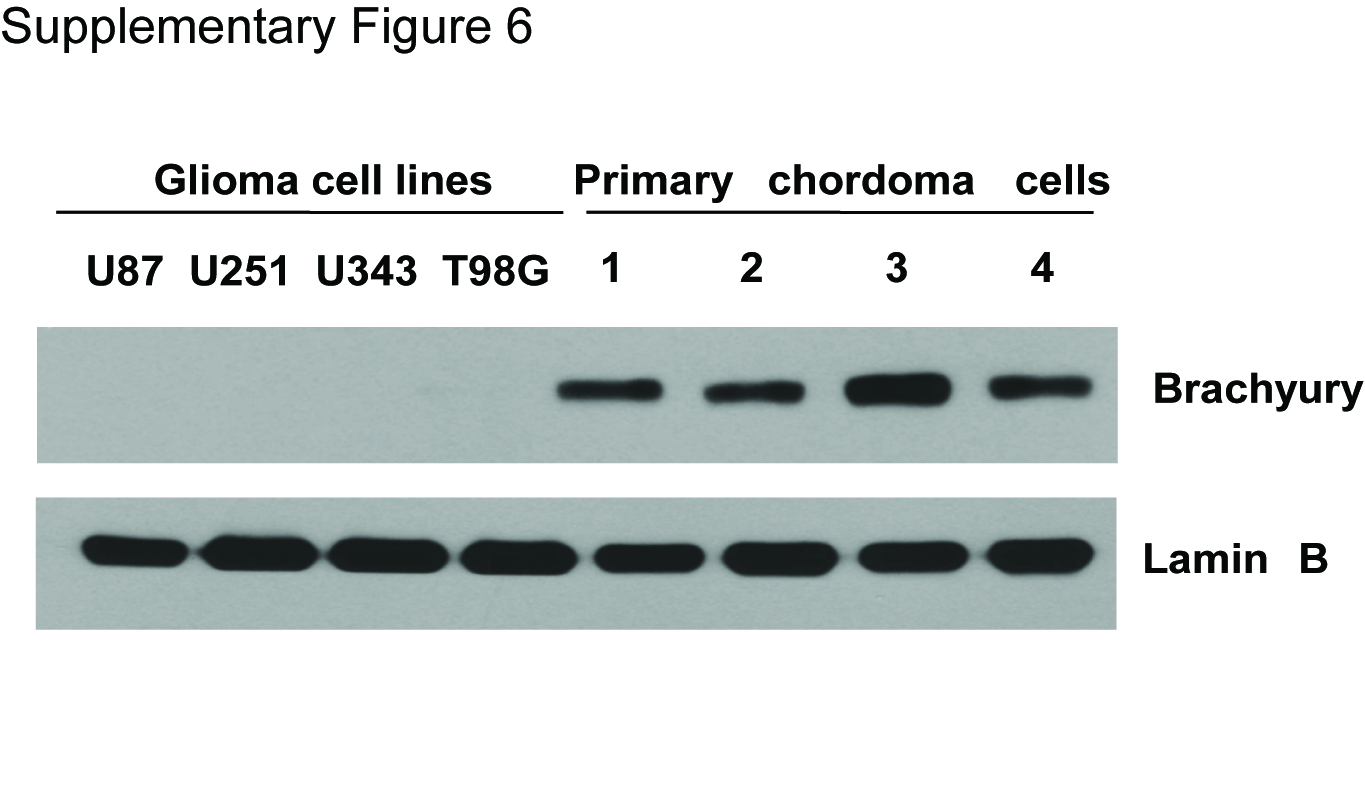

Supplement: S6 Fig — Immunoblot was performed on isolated nuclear protein fraction to demonstrate nuclear localization of brachyury that further supports immunohistochemical staining. (TIF) [file pone.0134426.s006.tif]
